# The cost of open heart surgery in Nigeria

**DOI:** 10.11604/pamj.2013.14.61.2162

**Published:** 2013-02-12

**Authors:** Bode Falase, Michael Sanusi, Adetinuwe Majekodunmi, Ifeoluwa Ajose, Ariyo Idowu, David Oke

**Affiliations:** 1 Cardiothoracic Division, Lagos State University College of Medicine, Lagos State University Teaching Hospital, Ikeja, Lagos, Nigeria; 2 Department of Anaesthesia, Lagos State University College of Medicine, Lagos State University Teaching Hospital, Ikeja, Lagos, Nigeria; 3 Adult Cardiology Unit, Department of Medicine, Lagos State University College of Medicine, Lagos State University Teaching Hospital, Ikeja, Lagos, Nigeria

**Keywords:** Open Heart Surgery, direct costs, Lagos, Nigeria

## Abstract

**Introduction:**

Open Heart Surgery (OHS) is not commonly practiced in Nigeria and most patients who require OHS are referred abroad. There has recently been a resurgence of interest in establishing OHS services in Nigeria but the cost is unknown. The aim of this study was to determine the direct cost of OHS procedures in Nigeria.

**Methods:**

The study was performed prospectively from November to December 2011. Three concurrent operations were selected as being representative of the scope of surgery offered at our institution. These procedures were Atrial Septal Defect (ASD) Repair, Off Pump Coronary Artery Bypass Grafting (OPCAB) and Mitral Valve Replacement (MVR). Cost categories contributing to direct costs of OHS (Investigations, Drugs, Perfusion, Theatre, Intensive Care, Honorarium and Hospital Stay) were tracked to determine the total direct cost for the 3 selected OHS procedures.

**Results:**

ASD repair cost $ 6,230 (Drugs $600, Intensive Care $410, Investigations $955, Perfusion $1080, Theatre $1360, Honorarium $925, Hospital Stay $900). OPCAB cost $8,430 (Drugs $740, Intensive Care $625, Investigations $3,020, Perfusion $915, Theatre $1305, Honorarium $925, Hospital Stay $900). MVR with a bioprosthetic valve cost $11,200 (Drugs $1200, Intensive Care $500, Investigations $3040, Perfusion $1100, Theatre $3,535, Honorarium $925, Hospital Stay $900).

**Conclusion:**

The direct cost of OHS in Nigeria currently ranges between $6,230 and $11,200. These costs compare favorably with the cost of OHS abroad and can serve as a financial incentive to patients, sponsors and stakeholders to have OHS procedures done in Nigeria.

## Introduction

Open heart Surgery (OHS) is not commonly performed in Nigeria. It was initially introduced to the country in 1974 and developed gradually but OHS activity in the only active centre at the time ceased by the year 2000 for various reasons, paramount of which was funding constraints [1–3]. There is a great disparity between the burden of cardiovascular disease and the surgical resources available in Nigeria. Both congenital and acquired heart diseases have a high prevalence. 25% of admissions of children in heart failure are due to congenital heart disease which is surgically correctable [4]. After hypertensive heart disease (56.7%), rheumatic heart disease (3.7%) is the most prevalent cardiac condition in adult patients referred for echocardiography [5]. 44% of medical admissions to a major Nigerian Teaching Hospital have been reported to be due to heart failure, of which 6.6% were due to rheumatic heart disease [6]. In the paediatric age group the commonest structural heart diseases in children referred for echocardiography in Lagos has been shown to be ventricular septal defect (41.7%) and atrial septal defect (20.2%) both of which are eminently correctable by surgery [7]. There is also a rising incidence of ischaemic heart disease [8, 9]. There is therefore a pressing need to establish the necessary resources to make OHS available in Nigeria which has already been done successfully in some West African cardiac centres [10, 11]. Efforts to resuscitate OHS in Nigeria are ongoing. 18 cases have been reported from Northern Nigeria [12] and more recently our group at the Lagos State University Teaching Hospital has reported 51 cases of OHS done between 2004 and 2011[13]. A number of other teaching hospitals are at various stages of preparation for starting OHS programs. The Lagos State Government has also recently completed a new Cardiac Centre which will likely commence activity in 2013.

Establishment and sustainability of OHS programs requires a lot of investment in material and human resources and the Achilles&apos heel of most programs in developing countries is financing of operations since the majority of the local populations are severely indigent [14–16]. Unlike developed countries, health insurance schemes are not well developed so financing of OHS is done by Governments, charitable organizations, and out of pocket payments by patients and their sponsors. There is great variability in the cost of OHS worldwide as shown by a comparative analysis of OHS costs by India Brand Equity Foundation [17]. This showed that Coronary Artery Bypass Grafting which costs $7000 in India costs $43,000 in the UK and $70,000-130,000 in the USA. The lack of OHS facilities in Nigeria has contributed to the drain on the Nigerian economy where an estimated $500 million is lost annually as over 5,000 Nigerians travel overseas for medical interventions at a cost of $20,000-$40,000 per traveler [18]. A recent World Bank report put the Gross Domestic Product per capita income of Nigeria at just $1,248 as compared to the United Kingdom with a Gross Domestic Product per capita of $35,686 [19] which emphasizes the fact that most Nigerian patients (apart from the privileged few) struggle to afford the cost of OHS and the current medical tourism is a huge drain on the economy that Nigeria can ill-afford.

It is therefore imperative that as OHS facilities are being developed in Nigeria the issue of financing is addressed, especially the issue of keeping costs low to make OHS affordable in Nigeria. This will however not be possible without initially determining the current cost of OHS in Nigeria which is unknown. The aim of the study was therefore to determine the current direct cost of OHS to assist Cardiac Surgery stakeholders in developing the resources for funding of OHS in Nigeria.

## Methods

### Institutional setting

Prior to November 2011, 48 OHS cases had been done in our institution. The procedures done were Mitral Valve Replacement in 14 patients (29.2%), Atrial Septal Defect Repair in 13 patients (27.1%), Ventricular Septal Defect Repair in 8 patients (16.7%), Aortic Valve Replacement in 5 patients (10.4%), excision of Left Atrial Myxoma in 2 patients (4.2%), Bidirectional Glenn Shunt in 2 patients (4.2%), Tetralogy of Fallot repair in 2 patients (4.2%), Coronary Artery Bypass Grafting in 1 patient (2.1%) and Mitral Valve Repair in 1 patient (2.1%).

### Surgical practice

Open Heart Surgery in our institution is performed in a designated facility called a Critical Care Unit (CCU). The CCU has 1 operating theatre, 4 intensive care beds, 4 single side rooms, a side laboratory for point of care testing and storage of blood products as well as a store for all the required cardiac hardware and consumables.

The core Cardiac Surgical Team is made up of 2 Cardiac Surgeons, 1 Cardiac Anaesthetist, 1 Intensivist, 1 Perfusionist, 6 theatre nurses and 8 intensive care nurses. This team is supported by 2 Adult Cardiologists, 1 Paediatric Cardiologist and 1 cardiac physiologist. Further support is provided by staffs in hematology, biochemistry, blood bank, central sterilization department and engineering (for supply of electrical power). Following patient referral an assessment is done to confirm the diagnosis. Part of this assessment is to assess the risk score (euroscore) as we currently try to avoid high risk cases at this stage of development of our program. The estimated cost of surgery is then discussed and a billing sheet (breakdown of the costs) is given to the patient who then goes on the waiting list until funds are raised for surgery. Once a group of two or three patients has confirmed having raised the funds for surgery they are batched together and worked up together for serial surgery, usually over 4-5 concurrent days. This is done to pool the resources of our institution to facilitate the surgery. Preoperative workup for all patients includes dental check (valve patients), transthoracic echocardiogram, chest radiogram, pulmonary function test, electrocardiogram, complete blood count, electrolytes, urea, creatinine, liver function tests, clotting profile, blood genotype, screening for human immunodeficiency virus and hepatitis B and C. For patients over 40 year of age or those in whom further information not available from transthoracic echocardiogram is required, referral is done to a private facility in Lagos [20] for cardiac catheterization. Our preference is to have coronary angiograms on all patients over 40 years to exclude concomitant coronary artery disease that would need to be addressed. Preparation for surgery involves cross matching and preparation of 4 units each of packed cells, fresh frozen plasma and platelets for each patient as we have found it prudent to prepare blood products in anticipation of bleeding rather than wait to source for blood products if required for postoperative bleeding.

For surgery requiring Cardiopulmonary bypass (CPB) our usual setup involves the use of the SC Compact Heart lung machine, a customized tubing pack with venous reservoir and oxygenator (Sorin, Italy), 21 Fr arterial cannula (Medtronic, USA), 28Fr and 32 Fr single stage venous cannulae (Medtronic, USA). Intermittent antegrade cold blood cardioplegia is used with cooling to 32 degrees centigrade.

### Cost categories

An initial pilot study was performed in June 2011 to capture the costs of 2 patients who underwent repair of Ventricular Septal Defects (VSD). A Microsoft Access Database was used to prepare a cost estimate of surgery via a billing sheet which is given to patients or their sponsors to raise the funds for surgery. Following the pilot study, 7 cost categories were identified as contributing to the direct cost of surgery. These were; (1) Investigations, (2) Drugs, (3) Theatre, (4) Perfusion, (5) Intensive care, (6) Honorarium and (7) Hospital Stay. Separate billing sheets were developed for each cost category and were used to capture the costs of all items used. All items used were counted prior to use and once used broken ampoules or packets were retained for postoperative reconciliation to verify the numbers of items used. This methodology to capture costs was tested successfully for the pilot study on the 2 VSD operations.

### Patient selection

As surgery for Atrial Septal Defect repair (ASD) and surgery for Mitral Valve Replacement (MVR) were the 2 most common operations, these procedures were selected as cases for cost determination. In addition there is a rising incidence of Ischemic Heart Disease in Nigeria and now that cardiac catheterization facilities are available in Lagos [20], more patients will be referred for Coronary Artery Bypass Grafting surgery (CABG). Costing of CABG was therefore also selected. Costing of these 3 procedures would therefore give a fair indication of the direct costs of the range of common OHS procedures performed in our program.

The study was performed prospectively between November 2011 and December 2011. From our waiting list, 1 case each for ASD, MVR and CABG was selected and worked up for surgery. Transthoracic echocardiogram in all the cases confirmed good left ventricular function and the absence of pulmonary hypertension. The ASD patient was a 20 year old male student with a euroscore of 2. The MVR patient was a 57 year old female teacher who was in good health, had no comorbidities and had a euroscore of 3. The patient for OPCAB was a 57 year old female teacher with unstable angina, two previous coronary stents, hypertension, peripheral vascular disease and poorly controlled diabetes. She was therefore a higher risk for surgery with a euroscore of 6. As she required only a graft to the left anterior descending artery it was decided to perform this case off bypass so the CABG case cost estimation was changed to that of Off Pump Coronary Artery Bypass Grafting (OPCAB).

### Collation of costs

Collation of costs was done by the predetermined cost categories. The costs are given in US Dollars (USD), converting from Nigerian currency (Naira) at the prevailing rate of exchange at the time of the study which was 162 Naira to 1 USD. For the various cost categories the USD value was used rounded off to one decimal point to maintain accuracy of costing. When totaling the overall costs the USD cost was rounded off to the nearest 5 dollars to allow conversion back to Naira which would more accurately reflect actual costs of Naira transactions when purchasing the various items as unit values of Naira are not used in every day transactions due to inflation.

Permission was obtained from the Institutional Ethics Committee for use of the existing patient data from the database. The results are presented below. Data analysis was done with Microsoft Excel 2010. Data is expressed as numbers or percentages, as appropriate.

## Results

### Surgical results

CPB was used for both the ASD repair and MVR. At surgery for the ASD repair a 4cm secundum defect was repaired with a native pericardial patch. For the MVR a stenosed rheumatic valve was excised and replaced with a 27mm St Jude Biocor Mitral valve. The OPCAB was performed using an Octopus 3 stabilizer system without priming a CPB circuit. The Left Internal Mammary Artery was anastomosed to the Left Anterior Descending Coronary Artery. All patients were transferred to the intensive care unit on minimal inotropic support which was weaned off within a couple of hours. All patients were extubated within 3 hours of surgery. Postoperative complications seen were readmission of both the ASD and OPCAB patients to the Intensive care unit on day 4. The OPCAB patient had a chest infection which required antibiotic treatment and a left sided pleural effusion which required tube drainage. In addition she was fluid overloaded, which responded to diuresis. The ASD patient also had a chest infection as well as a pleural effusion that required chest tube drainage. The MVR patient went into atrial fibrillation on day 4 which was successfully treated and cardioverted with Amiodarone. The ASD patient was discharged home from the CCU after 7 days but CCU stay was longer in the MVR and OPCAB patients due to their complications (Table 1).

**Table 1:** Patient details

Details	ASD	MVR	OPCAB
Age (years)	20	57	56
Sex	Male	Female	Female
Euroscore	2	3	6
CPB time (mins)	60	122	Not applicable
Cross clamp time (mins)	30	108	Not applicable
Ventilation time (hours)	2	3	3
Postoperative Blood loss (mls)	793	494	483
Packed cells transfused(units)	2	2	2
ITU stay (days)	2	2	4
Hospital stay (days)	7	11	10
Complications	Chest infection Pleural effusion	Atrial fibrillation	Fluid overload Chest infection Pleural effusion

ASD: Atrial Septal Defect; MVR: Mitral Valve Replacement; OPCAB: Off Pump Coronary Artery Bypass Grafting

### Cost Categories

**Investigations**: The cost of investigations was ASD $955, MVR $3040 and OPCAB $3020 (Table 2):

**Drugs**: The cost of drugs was ASD $600, MVR $1200 and OPCAB $740 (Table 3)

**Theatre**: The theatre costs were ASD $1,360, MVR $3,535 and OPCAB $1305 (Table 4)

**Perfusion**: The perfusion costs were ASD $1,080, MVR $1,100 and OPCAB $915 (Table 5).

**Intensive care**: The cost of intensive care treatment was ASD $410, MVR $500 and OPCAB $625 (Table 6).

**Honorarium**: The cost of Honorarium was constant for all cases at $925.

**Hospital stay**: The cost of hospital stay was constant for all cases at $900.

**Total cost of OHS procedures**: The ASD cost was the lowest at $6,230 followed by the OPCAB cost of $8,430. The highest cost was seen with the MVR at $11,200 (Table 7).

**Table 2:** Investigations performed and cost (in USD)

Item	Unit Cost	ASD Qty	ASD Cost	MVR Qty	MVR Cost	OPCAB Qty	OPCAB Cost
Arterial Blood Gas Cartridge	27.8	11	305.8	15	417.0	15	417.0
Blood group	6.2	1	6.2	1	6.2	1	6.2
Genotype	12.4	1	12.4	1	12.4		
HIV and Hepatitis Screen	66.7	1	66.7	1	66.7	1	66.7
Pulmonary function Test	6.2	1	6.2	1	6.2		
Chest Xray	12.4	1	12.4	1	12.4	1	12.4
Echocardiogram	61.8	1	61.8	1	61.8	1	61.8
12 lead Electrocardiogram	6.2	1	6.2	1	6.2	1	6.2
Electrolytes, Urea, Creatinine	22.8	1	22.8	1	22.8	1	22.8
Liver function Tests	30.9	1	30.9	1	30.9	1	30.9
PT/INR	18.5	1	18.5	1	18.5	1	18.5
Full blood count	18.5	1	18.5	1	18.5	1	18.5
Blood Products	385.8	1	385.8	1	385.8	1	385.8
Cardiac Catheterization	1,975.3			1	1,975.3	1	1,975.3
**TOTAL COST**			**954.2**		**3,040.7**		**3,022.1**

ASD: Atrial Septal Defect; MVR: Mitral Valve Replacement; OPCAB: Off Pump Coronary Artery Bypass Grafting, Qty: Quantity

**Table 3:** Drugs used and cost (in USD)

Item	Unit Cost	ASD Qty	ASD Cost	MVR Qty	MVR Cost	OPCAB Qty	OPCAB Cost
Dopamine 200mg/5ml IV	16.0	**2**	32.0	3	48.0	3	48.0
Amiodarone 150mg/3ml IV	7.1			60	426		
Atropine 600mcg/ml IV	0.9	1	0.9	3	2.8	3	2.8
Calcium gluconate 10%IV	4.3	1	4.3	3	12.9	1	4.3
Cardioplegia 20ml IV	9.9	**2**	19.8	4	39.6		
Glycopyrronium 0.2mg/ml IV	3.1	**2**	6.2	3	9.3	2	6.2
Heparin 5000IU/5ml IV	8.6	**2**	17.2	3	25.8	1	8.6
Fentanyl 50mcg/ml IV	6.2	**1**	6.2	2	12.4	1	6.2
Magnesium Sulphate 5g/10ml IV	4.3			2	8.6		
Midazolam 10mg/2ml IV	4.3	**1**	4.3	1	4.3	1	4.3
Nitroglycerin 5ml/ml IV	50.6	**3**	151.8	1	50.6	3	151.8
Noradrenaline 1mg/ml IV	14.2			2	28.4	1	14.2
Potassium Chloride 20mmol/10ml IV	1.3			4	5.2	3	3.9
Propofol 10mg/ml IV	7.1	**4**	28.4	4	28.4	4	28.4
Protamine Sulphate 50mg/5ml IV	5.6	**5**	28.0	12	67.2	4	22.4
Suxamethonium 100mg/2ml IV	1.2	**2**	2.4	2	2.4	2	2.4
Tranexemic Acid 100mg/ml IV	4.9	**2**	9.8	2	9.8	2	9.8
Vecuronium 10mg IV	18.5	**2**	37.0	3	55.5	2	37.0
Phenylephrine 10mg/ml IV	12.4	**1**	12.4	1	12.4	1	12.4
Sodium Bicarbonate 840mg/10ml IV	1.5	**3**	4.5	6	9.0		
Isoflurane	15.4	**1**	15.4	1	15.4	1	15.4
Cefriaxone 1g IV	18.5	**10**	185.0	10	185.0	10	185.0
Lasix 20 mg IV	1.5	**4**	6.0	19	28.5	4	6.0
Paracetamol 1g IV	0.6	**4**	2.4	3	1.8	2	1.2
Tramadol 50mg IV	1.9					1	1.9
Omeprazole 20mg IV	13.9			2	27.8	2	27.8
Diclofenac 75mg IV	1.9	**1**	1.9	3	5.7	2	3.8
Insulin IV	1.9					2	3.8
Ondansetrone 2mg/ml IV	1.9	**1**	1.9			1	1.9
Slow K 600mg tab	0.3	**1**	0.3	5	1.5	9	2.7
Moduretic 20mg tab	0.6			4	2.4		
Paracetamol 500mg tab	0.2	**6**	1.2	44	8.8	48	9.6
Diclofenac tab 50 mg	1.2			3	3.6	11	13.2
Tramadol 50mg tab	1.9	**12**	22.8	12	22.8	12	22.8
Aspirin 75 mg	0.6			28	16.8	52	31.2
clopidogrel 75 mg	1.9			8	15.2	10	19.0
Coartem tab	0.3					8	2.4
Amlodipine 10 mg tab	1.2					6	7.2
Omeprazole 20mg tab	0.9					13	11.7
simvastatin 20 mg tab	0.4			4	1.6	28	11.2
Metoprolol 50 mg tab	1.5	**2**	3.0	1	1.5	1	1.5
Amiodarone 50 mg tab	0.6			4	2.4		
**Total cost**			**602.1**		**1199.4**		**742.0**

**ASD: Atrial Septal Defect; MVR:** Mitral Valve Replacement; OPCAB: Off Pump Coronary Artery Bypass Grafting, Qty: Quantity

**Table 4:** Theatre Items used and cost (in USD)

Item	Unit Cost	ASD Qty	ASD Cost	MVR Qty	MVR Cost	OPCAB Qty	OPCAB Cost
Chest Tube	18.5	3	55.6	3	55.6	3	55.6
Pleurevac	55.6	2	111.1	2	111.1	2	111.1
Urine Bag	24.7	1	24.7	1	24.7	1	24.7
Heart Surgery Drapes	86.4	1	86.4	1	86.4	1	86.4
Endotracheal Tube	15.4	1	15.4	1	15.4	1	15.4
Ligaclip	2.5	1	2.5	3	7.4	4	9.9
Pacing wires	18.5	1	18.5	2	37.0	2	37.0
Mitral SJM Biocor Valve	1975.3			1	1,975.3		
Yankauer Suction	7.4			2	14.8		
Suture Monocryl 3/0	4.3			2	8.6	2	8.6
Suture Prolene 3/0	3.1	6	18.5	2	6.2		
Suture Prolene 4/0	6.2	5	30.9	5	30.9		
Suture Prolene 7/0	8					2	16.0
Suture Vicryl 1	4.3	2	8.6	1	4.3		
Suture Vicryl 2/0	3.7			1	3.7	3	11.1
Silk 2-0	0.3	2	0.6	3	0.9	3	0.9
Silk 2	1.5	6	9.3	3	4.6	4	6.2
Umbilical Tape	4.9	2	9.9	1	4.9		
sternal Wires	30.9	1	30.9	1	30.9	1	30.9
Pledgets	18.5	1	18.5	1	18.5	1	18.5
Vascular Tourniquet	30.9	1	30.9	1	30.9		
Suture Ethibond 2-0 Pledgetted	98.8			2	197.5		
Suture Ethibond 2-0 Single	7.4	3	22.2				
Hotline Fluid Warming Set	18.5	1	18.5	1	18.5	1	18.5
Luer LOK 50ml Syringe	3.1	5	15.4	4	12.3	7	21.6
Shoe Cover	15.4	1	15.4	1	15.4	1	15.4
Masks	15.4	1	15.4	1	15.4	1	15.4
Nurses cap	15.4	1	15.4	1	15.4	1	15.4
Surgical Gowns	9.9	6	59.3	6	59.3	6	59.3
Surgical Blades	1.5	5	7.7	5	7.7	5	7.7
Laparotomy pack	1.5	15	23.1	15	23.1	15	23.1
Raytec Gauze	0.9	15	13.9	15	13.9	15	13.9
Plain Gauze	0.8	30	23.1	30	23.1	30	23.1
Sterile Surgical Gloves	3.1	14	43.2	14	43.2	14	43.2
Examination Gloves (1 box)	61.7	1	61.7	1	61.7	1	61.7
Diathermy Cable	30.9	1	30.9	1	30.9	1	30.9
Diathermy Pad	30.9	1	30.9	1	30.9	1	30.9
Compressed Air Cylinder	123.5	2	246.9	2	246.9	2	246.9
Oxygen Cylinder	123.5	2	246.9	2	246.9	2	246.9
**Total**			**1,362.2**		**3,534.6**		**1,306.5**

ASD: Atrial Septal Defect; MVR: Mitral Valve Replacement; OPCAB: Off Pump Coronary Artery Bypass Grafting, Qty: Quantity

**Table 5:** Perfusion Items used and cost (in USD)

Item	Unit Cost	ASD Qty	ASD Cost	MVR Qty	MVR Cost	OPCAB Qty	OPCAB Cost
ACT Cuvette	6.8	4	27.2	5	34	5	34
Cannula Venous Right Angled Metal 24Fr	43.2	1	43.2	1	43.2		
Cannula Venous Right Angled Metal 31Fr	43.2	1	43.2	1	43.2		
Cannula Aortic Curved 18fr	40.1	1	40.1	1	40.1		
Cannula Aortic Root with Vent Line 14 Ga	14.2	1	14.2	1	14.2		
Connector Straight 1/2 X 3/8	4.3					2	8.6
Connector Y 1/4 x 3/8x 3/8	6.2	2	12.4	2	12.4	1	6.2
Set PCP 5 Fr	34.6	1	34.6	1	34.6		
OPCAB Stabilizer	864					1	864
Suction LV Vent 16 Fr	14.2			1	14.2		
Perfusion Pack	740.7	1	740.7	1	740.7		
Cardioplegia pack	123.5	1	123.5	1	123.5		
**Total**			**1,079.1**		**1,100.1**		**912.8**

ASD: Atrial Septal Defect; MVR: Mitral Valve Replacement; OPCAB: Off Pump Coronary Artery Bypass Grafting, Qty: Quantity

**Table 6:** Intensive care items used and cost (in USD)

Item	Unit cost	ASD Qty	ASD Cost	MVR Qty	MVR Cost	OPCAB Qty	OPCAB Cost
Tegaderm (IV Dressing)	3.1	1	3.1	3	9.3	2	6.2
T Plus 8 x 10cm (Wound Dressing)	1.2			2	2.4		
T Plus 8 x 15cm (Wound Dressing)	1.9	2	3.8				
T Plus10 x 25cm (Wound Dressing)	2.5	3	7.5	3	7.5	3	7.5
Dressing pack	1.5	5	7.5	8	12.0	5	7.5
Gelofusine 500ml bag	29.1	3	87.3	5	145.5	4	116.4
Normal Saline 500ml bag	1.9	2	3.8	5	9.5	4	7.6
Normal Saline (Cartoon)	37.0	1	37.0	1	37.0	1	37.0
Anti-embolism Stocking	21.6	1.0	21.6	1	21.6	1	21.6
Suction catheter	6.2	3	18.6	3	18.6	6	37.2
Foley Catheter	6.2	1	6.2	1	6.2	1	6.2
Urine bag	1.9	1	1.9	1	1.9	1	1.9
Arterial catheter 20Ga	37.0	1	37.0	1	37.0	1	37.0
Central Venous Catheter 7 Fr	92.6	1	92.6	1	92.6	1	92.6
Swan-Ganz Catheter 7.5 Fr	154.3					1	154.3
Monitoring Lines 150cm m/f	3.1	2	6.2	4	12.4	4	12.4
Monitoring Lines 50cm m/f	2.5	1	2.5				
Transducer Double Channel	49.4	1	49.4	1	49.4	1	49.4
Three way tap (with extension)	3.1	4	12.4	5	15.5	4	12.4
IV Giving Set	1.9	1	1.9	4	7.2	4	7.2
Venous Cannula 16G	0.6	1	0.6	3	1.8	2	1.2
Blood giving set	1.9	2	3.8	2	3.8	1	1.9
50 ml Syringe	0.6	2	1.2	2	1.2	2	1.2
Syringe and needle	0.2	26	5.2	30	6.0	30	6.0
**Total cost**			**411.1**		**498.4**		**624.7**

ASD: Atrial Septal Defect; MVR: Mitral Valve Replacement; OPCAB: Off Pump Coronary Artery Bypass Grafting, Qty: Quantity

**Table 7:** Total cost of Open Heart Surgery Procedures (in USD and Naira)

Category	ASD (USD)	ASD (NAIRA)	MVR (USD)	MVR (NAIRA)	OPCAB (USD)	OPCAB (NAIRA)
Drugs	600	97,200	1200	194,400	740	119,880
Intensive Care	410	66,420	500	81,000	625	101,250
Investigations	955	154,700	3,040	492,480	3020	489,240
Perfusion	1,080	174,960	1,100	178,200	915	148,230
Theatre	1,360	220,320	3,535	572,670	1305	211,400
Honorarium	925	150,000	925	150,000	925	150,000
Hospital Stay	900	145,000	900	145,000	900	145,000
**Total (USD)**	**6,230**	**1,008,600**	**11,200**	**1,813,750**	**8,430**	**1,365,000**

ASD: Atrial Septal Defect; MVR: Mitral Valve Replacement; OPCAB: Off Pump Coronary Artery Bypass Grafting, Qty: Quantity. Naira is Nigerian currency, USD is US Dollars

## Discussion

OHS activity has remained very low in Nigeria. Our institution is currently the only one in the country with any intermittent OHS activity. Between August 2004 and December 2011 only 51 OHS procedures have been performed, with 8 mortalities (15.7%). The results would be improved with a higher volume of cases, but the annual number of cases has remained low, in part because of the cost implication of OHS. The causes of a low total number of cases done annually are multifactorial. Other important factors apart from cost include the level of public awareness of the existence of such a programme locally, established referral lines between cardiologists as well as corporate sponsors with trusted institutions abroad, number of cases done which can be directly interpreted as the experience of the institution, mortality and morbidity data and the level of public confidence in the institution.

**Figure 1:**
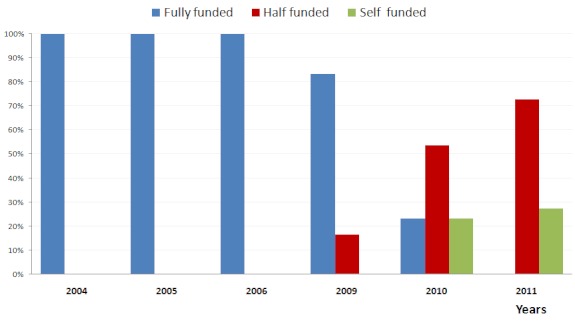
Annual level of funding of Open Heart Surgery by Government. Full funding - Government covered entire cost of surgery. Half funding- Government covered half the cost, patient and sponsors made up the difference. Self-funding - No Government funding. Procedure fully funded by patient or sponsors

Between 2004 and 2006 all the cases done were fully funded by the Lagos State Government. Since 2009 due to economic exigencies there has been a decline in the number of cases the Government has been able to fund and a rise in the number of cases that are partly or fully funded by patients or their sponsors (Figure 1). Ironically the paucity of funding for OHS does not mean there is lack of a need for such surgery, as seen from the numbers of Nigerian patients that travel abroad for medical tourism [18] as well as the number of patients on the waiting list for OHS. When this study was performed, there were 102 patients on the waiting list. Some of these patients have been waiting for sponsorship for greater than one year. It has been shown that only 15% of patients in another West African Centre are able to raise funds within a year of diagnosis [21]. The few patients in our programme able to raise the funds for surgery average 3-6 months to do so. As efforts continue to develop programs for OHS in Nigeria, the first step is to determine the local cost of surgical intervention, which was the purpose of this study. This study has shown that the current costs of OHS in our institution are $6,230 for repair of Atrial Septal Defect, $8,430 for Off Pump Coronary Artery Bypass Surgery, and $11,200 for Mitral Valve Replacement Surgery.

### Reasons for differences in the cost categories

**Investigations**: At $3040 and $3020 respectively, the cost of investigations for the MVR and OPCAB were similar. The ASD only cost $955 because being a young man under the age of 40 years with no history suggestive of myocardial ischaemia it was not necessary to perform a coronary angiogram. The Cardiac catheterization cost of $1,975 considerably increased the investigation costs for the MVR and OPCAB (Table 2).

**Drugs**: The MVR drugs cost was the highest at $1200, compared to $740 for the OPCAB and $600 for the ASD (Table 3). The increased expense for the MVR was due to the use of Amiodarone to control the complication of atrial fibrillation which occurred in the MVR.

**Theatre**: The MVR was the most expensive at $3,535 as compared to the ASD of $1,360 and OPCAB of $1305. This was due to the use of the use of a bioprosthetic heart valve for the MVR which cost $1975 (Table 4).

**Perfusion**: With a cost of ASD $1,080, MVR $1,100 and OPCAB $915 the perfusion costs were quite similar for the three procedures. Though the ASD and MVR required the use of a perfusion pack and cardioplegia pack which was not used for the OPCAB, the savings that could have accrued for the OPCAB was offset by the cost of the Octopus 3 stabilizer system used for the OPCAB (Table 5).

**Intensive care**: The cost of intensive care treatment was ASD $410, MVR $1400 and OPCAB $1525. The differences in cost are accounted for by both the MVR and OPCAB requiring more Gelofusine (Colloid Infusion) and in addition a Swan-Ganz Catheter was used for the OPCAB patient (Table 6).

**Honorarium**: At present personnel costs are covered by a small honorarium of $925 which is paid as part of the direct cost of OHS. This is much lower than the operating fee of $1,400 paid to staff in the Netherlands [22] and apart from this honorarium there are no further incentives. As OHS is further developed incentives like assistance with mortgage facilities, car loans and increased remuneration need to be developed, otherwise the grass will always look greener on the other side. A brain drain of trained cardiac staff occurred in the early years of the program in Ghana [10] and was addressed by developing some of the incentives mentioned above.

**Hospital stay**: Hospital costs are to a large extent subsidized by the hospital to encourage growth of the OHS programme. The cost of hospital stay in the CCU that would otherwise apply is as follows. Ventilated patients cost $310 daily, non-ventilated patients on monitored beds cost $155 daily and stay in the non-monitored side rooms in CCU costs $60 daily. To keep costs down the hospital management agreed to a package cost of $900 for the cost of hospital stay. In return the cardiac team works towards fast-tracking patients where possible to minimize their CCU stay. An option now available (which will increasingly be used) is early transfer to the Cardiothoracic Open Ward where the cost of hospital stay for a week is only $45.

### Comparison of costs with other Cardiac Centres

These costs compare favorably with costs seen with other cardiac programs in Africa and India and are much lower than the costs in Europe and the USA. At the National Cardiothoracic Centre in Ghana the average cost of OHS is quoted as $5000 for Ghanaians as the Ghanaian Heart Foundation often subsidizes 50% of the cost for Ghanaian patients [10]. In Kenya costs for OHS were noted to vary between the public sector and private sector (personal communication). 86 Kenyan Shillings (Khs) is one USD. In the public sector (Kenyatta Hospital) patients are required to raise 100,000 Khs following which the surgery is done with the government bearing the remaining cost. In the private sector in Kenya, 4 hospitals (Aga Khan University Teaching Hospital, Karen Hospital, Nairobi Hospital and Mater Hospital) offer OHS at a package cost (with or without a cardiac prosthesis) ranging from 600 - 920,000 Khs. The cost of surgery in Kenya therefore currently varies between $7000 and $10,700. In Uganda the Mulago Hospital offers OHS at a package cost of $5000 (personal communication). The only previous publication from Nigeria which referred to the cost of OHS was from Enugu [2] and during the period reviewed (1974-2000) the cost of OHS was quoted as $4,800 without a prosthesis and $5,600 with prosthesis. These costs are however over a decade old.

### Potential for cost reduction

Our MVR and OPCAB costs at $11,200 and $8,430 respectively were considerably more expensive than ASD surgery. A number of factors contributed to this. For both procedures Cardiac Catheterization was required and as this is currently performed in a private hospital in Lagos we have no control over the cost which at almost $2000 is very high. Comparatively, Cardiac catheterization in India can cost as little as $100. It is hoped that when the Lagos State Government opens a new Cardiac Centre due to be commissioned in 2013 the cost of cardiac catheterization will be considerably reduced. An additional cost was the cost of the bioprosthetic valve used for the MVR ($1975). This was purchased from South Africa and till this cost is reduced it will increase the cost of MVR in our environment.

An area further cost savings may obtain in future is in re-sterilization of cardiac consumables with ethylene oxide which has been shown to be safe in a number of studies [23–25]. This is yet to be explored as there is a need to establish the safety of this approach in our environment as well as raise the funds to purchase the necessary equipment. If initiated, it could result in significant cost savings as volumes of surgery increase. As more Cardiac Units are established to offer OHS in Nigeria it is anticipated that consumables required can be bulk purchased and more favorable terms negotiated to reduce the costs.

### Study limitations

1. The risk profile of each patient was different, with the highest risk being the OPCAB patient with a euroscore of 6 as compared to a euroscore of 2 for the ASD patient and a euroscore of 3 for the MVR patient. It is likely that if a lower risk patient had been selected for the OPCAB the costs would have been reduced. It has been shown that selection of patients with lower risk profiles reduces intensive care stay and hospital stay, with resulting cost savings. Euroscore can therefore predict the direct costs of surgery with a difference of $2000 between a patient of euroscore 0-2 and a patient with euroscore 5-6 [26]. However the higher risk profile of the OPCAB patient is increasingly seen in patients with ischemic heart disease who present for surgery so the cost obtained could be representative of the typical cost for coronary surgery in our environment.
2. The cost for the ASD would have been increased in an older patient who would have required cardiac catheterization so the cost obtained reflects the cost of surgery for a young low risk ASD patient as opposed to an older, higher risk ASD patient. However apart from one case, all the 14 ASD surgeries done in our institution have been below the age of 40 years so this cost is representative of our practice.
3. The cost obtained for the OPCAB may not accurately reflect of the cost of surgery by conventional CABG using cardiopulmonary bypass. There is evidence from the literature that the cost differential of OPCAB and conventional CABG may be about 15%. In a study from the Netherlands the direct cost of CABG was $10,095 while that of OPCAB was$ 8,720, a difference of $1375, making OPCAB 13.6% cheaper than CABG [22]. We will therefore still need to make a determination of the cost of CABG as more of such cases accrue.
4. This study was limited to determining the direct cost of OHS. Additional costs may be incurred during follow up of the patient. In our environment these costs are borne by the patient and are yet to be determined, but it was shown that going by costs in 2003 the additional cost for CABG in the Netherlands when followed up to one year is an additional $5000 [21].
5. The cost determination for this study was limited to three procedures. Though some extrapolation is possible in assessing the cost of other OHS procedures, further costing of other specific OHS procedures needs to be done.

## Conclusion

This study has shown that the current direct cost of OHS at our institution ranges between $6,230 and $11,200. Future cost reductions may come from reduction in the costs of consumables, reduction of the cost of Cardiac Catheterization once the Government opens its own catheterization facility, reduction of the cost of cardiac prostheses and possibly in the use of ethylene oxide to re-sterilize consumables. A realistic target would be working towards reducing the cost of OHS by at least $1000 which would make OHS even more affordable for patients and their sponsors. Facilities for OHS have been developed at the Lagos State University Teaching Hospital, despite the constraints and challenges of our environment. With this study a benchmark for the current cost of OHS in Nigeria, as obtains in our institution, has been determined. Funding options need to be developed to fund OHS in Nigeria now that the cost of performing surgery locally has been determined. Without the development of funding options as obtains in some other successful programs in Africa the growth of OHS programs in Nigeria will remain stunted.
